# Transesophageal Echocardiographic Guidance of Transapical Aortic Cannulation for Cardiopulmonary Bypass

**DOI:** 10.1016/j.case.2026.03.008

**Published:** 2026-05-20

**Authors:** John Foster, Nicolas Pope, David Carroll

**Affiliations:** a MUSC Department of Anesthesiology and Perioperative Medicine, Charleston, South Carolina; b Division of Cardiothoracic Surgery, MUSC Department of Surgery, Charleston, South Carolina

**Keywords:** Transapical aortic cannulation, Acute type A aortic dissection cannulation strategies, Transesophageal echocardiography procedural guidance

## Abstract

• TAC for acute type A aortic dissection is shown.
• TEE is invaluable in the perioperative management of acute type A aortic dissection.
• TEE is essential for guiding aortic cannulation in acute type A aortic dissection.
• Overview of TAC and TEE guidance of procedure is given.

## Graphical abstract

**Figure:**
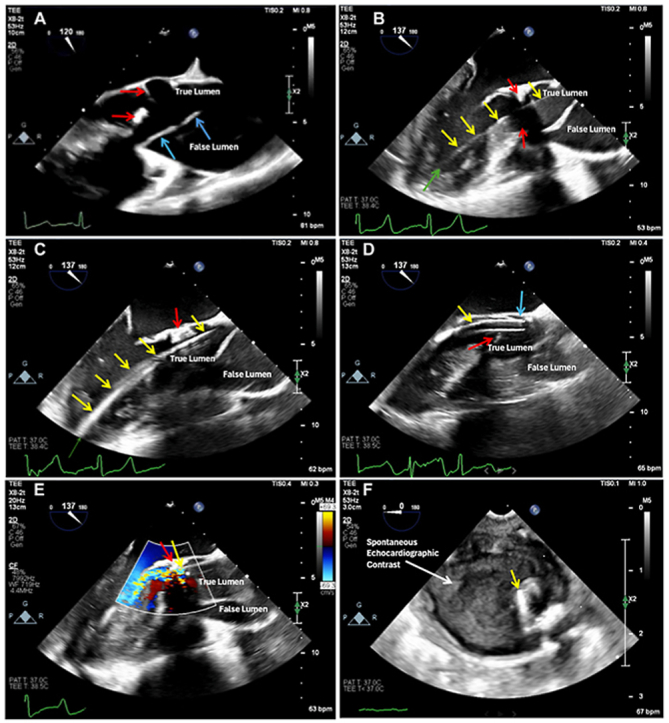

## Introduction

Transapical aortic cannulation (TAC) for cardiopulmonary bypass (CPB) has been described in cardiothoracic surgery literature as a rapid, reliable, and safe method for arterial cannulation during the surgical treatment of acute type A aortic dissection.^1^, ^2^, ^3^, ^4^, ^5^, ^6^

Transesophageal echocardiography (TEE) is an invaluable resource in the evaluation and management of patients with acute type A aortic dissection. Transesophageal echocardiography is used to confirm the diagnosis of acute type A aortic dissection, determine the need for aortic valve (AV) intervention, determine the extent of aortic dissection repair necessary, safely guide the cannulation procedure, and assist in hemodynamic monitoring throughout the perioperative period.^7^ With regards to TAC, experts strongly recommend using or routinely employing TEE to guide TAC.^1^^,^^3^^,^^5^^,^^6^ However, there are no available resources outlining the use of TEE to guide TAC and identify potential complications unique to TAC. This case report emphasizes the critical role of TEE in directing the TAC procedure and providing echocardiographers with the tools necessary to safely and effectively guide the procedure.

## Case Presentation

A 65-year-old woman with a medical history of a bicuspid AV presented with an acute onset of chest pain, right upper extremity weakness, and confusion. Computed tomography angiography (CTA) of the chest, abdomen, and pelvis demonstrated an acute type A aortic dissection that involved the innominate artery and bilateral carotid arteries and that extended distally to the bilateral femoral arteries (Figure 1). The patient was taken emergently to the operating room for surgical repair of the acute type A aortic dissection.

**Figure 1:**
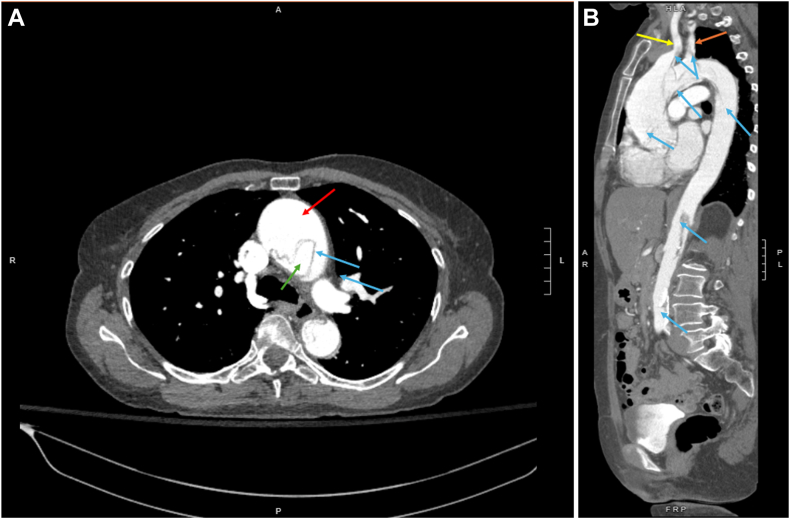
**(A)** Axial image from the CTA of the chest obtained on presentation demonstrates an ascending aortic aneurysm and acute type A aortic dissection with an anterior false lumen *(red arrow)*, posterior true lumen *(green arow)*, and intimal flap *(blue arrows)*. **(B)** Sagittal image from the CTA of the chest, abdomen, and pelvis obtained on presentation demonstrates an ascending aortic aneurysm and acute type A aortic dissection with intimal flaps *(blue arrow)* visible in the ascending aorta, aortic arch, descending aorta, innominate artery *(yellow arrow)*, and right carotid artery *(orange arrow)*.

Intraoperative TEE was notable for normal biventricular systolic function, bicuspid AV, moderate aortic stenosis (AS; AV area 1.1 cm^2^, mean pressure gradient 29 mm Hg, peak velocity 3.4 m/s), moderate aortic regurgitation (AR; vena contracta 4.5 mm), 54 mm ascending aorta aneurysm, and an acute type A aortic dissection extending from aortic root to descending thoracic aorta beyond visualization with TEE (Figure 2A, Video 1).

**Figure 2:**
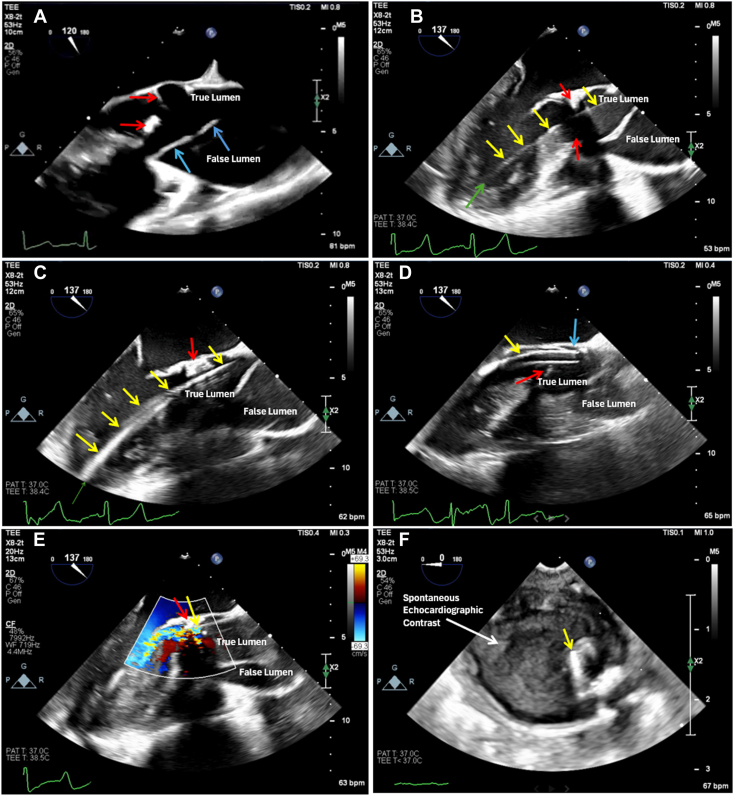
**(A)** Two-dimensional TEE, midesophageal long-axis (120°) view, demonstrates the bicuspid AV *(red arrows)*, dilated ascending aorta, and acute type A thoracic ascending aortic dissection with multiple mobile, linear tissues consistent with intimal dissection *(blue arrows)*. Two-dimensional TEE, midesophageal long-axis (137°) diastolic views without **(B–D)** and with color-flow Doppler **(E)**, demonstrates the transapically placed wire *(yellow arrows)* originating at the LV apex *(green arrow)*, crossing the AV *(red arrows)*, and progressively entering the true lumen of the ascending aorta **(B** and **C)** and terminating in the true lumen of the aortic root at the level of the sinus of Valsalva (**D**; *blue arrow*); AR is seen originating at the site where the aortic cannula *(yellow arrow)* crosses the AV *(red arrow*; **E**). **(F)** Two-dimensional TEE, transgastric midventricular short-axis (0°) view during ventricular fibrillation, demonstrates a distended left ventricle with dense spontaneous echo contrast and the transapical aortic cannula *(yellow arrow)*.

Initial arterial canulation attempts were performed on the bilateral femoral arteries; however, the true lumens of both femoral arteries were unable to be accessed. Because the initial CTA showed dissection involving the innominate artery, a right axillary/innominate cannulation technique was not attempted, and a left axillary artery cutdown was performed instead. However, exposure of the left axillary artery revealed evidence of dissection and the left axillary artery cannulation attempt was aborted. Central aortic cannulation was not attempted due to the presence of a small true lumen located on the lesser curve of the aorta that could not be safely accessed.

Given the inability to safely cannulate in this case via the previously mentioned sites, the decision was made to proceed with TAC. A left mini thoracotomy was performed to expose the left ventricular (LV) apex. After heparinization, a needle was passed through the LV apex through which a stiff wire was passed and guided across the AV and into the true lumen of the ascending aorta with TEE (Figure 2B, Video 2). An aortic cannula was then passed over the wire, and TEE guidance was used to position the cannula tip in the true lumen of the ascending aorta at the level of the sinus of Valsalva (Figure 2C and D, Videos 3 and 4).

After initiation of CPB, true lumen perfusion was confirmed, and the patient was cooled in anticipation of circulatory arrest. During the cooling period, an echocardiographic evaluation of the cannula and AV identified persistent AR (Figure 2E, Video 5). After the onset of hypothermic ventricular fibrillation, TEE examination was notable for unchanged AR and a distended left ventricle (Figure 2F). After vent placement in the main pulmonary artery, left atrial appendage, and left superior pulmonary vein, TEE confirmed satisfactory left heart decompression. Transesophageal echocardiography was used periodically to monitor for adequate LV venting, correct cannula positioning, and true lumen perfusion during the cooling phase leading up to circulatory arrest; however, no further intervention was required.

After the target temperature of 18 °C was reached, circulatory arrest was initiated by draining the circulating blood volume and clamping the arterial limb of the CPB circuit. Retrograde cerebral perfusion was delivered via a venous cannula in the superior vena cava. The surgeon then proceeded with transverse aortic arch replacement using an aortic graft with a side-arm branch and endovascular graft deployment distal to left subclavian artery. The arterial limb of the circuit was then moved to a side-arm branch on the aortic graft, and CPB was reinitiated. The surgeon then removed the transapical cannula and sutured the TAC site at the LV apex. Lastly, the surgeon proceeded with an AV replacement, aortic root replacement, and ascending aorta replacement with graft-to-graft anastomosis.

After weaning the patient from CPB, a complete TEE examination was performed, with emphasis on evaluating the bioprosthetic AV and native mitral valve, ensuring that the endovascular stent was deployed in the true lumen of the descending thoracic aorta and ensuring true lumen patency distal to the endovascular stent.

The patient was transferred to the intensive care unit in stable condition with an open chest due to coagulopathy. Delayed sternal closure was performed on postoperative day 2. A repeat CTA of the chest, abdomen, and pelvis was performed on postoperative day 3, demonstrating replacement of the AV, aortic root, ascending aorta, and aortic arch; endovascular stent deployment to the proximal descending thoracic aorta; and persistent dissection in the bilateral subclavian arteries and bilateral carotid arteries and inferior extension in the descending thoracic aorta to the level of the bilateral iliac arteries. Postoperative course was complicated by acute hypoxemic respiratory failure due to hospital-associated pneumonia. The patient was discharged home on postoperative day 29.

## Discussion

Common arterial cannulation sites for CPB in patients with an acute type A aortic dissection are femoral, axillary/innominate, carotid, and ascending aortic.^8^, ^9^, ^10^, ^11^, ^12^, ^13^ While still an uncommon technique, TAC is a valuable arterial cannulation strategy that has been described in the cardiothoracic surgery literature.^1^, ^2^, ^3^, ^4^, ^5^, ^6^ Each arterial cannulation method has potential advantages and disadvantages that must be considered, including those unique to TAC (Table 1).^8^, ^9^, ^10^, ^11^, ^12^, ^13^

**Table 1:** Summary of the advantages and disadvantages of the TAC technique and other common arterial cannulation techniques in the setting of acute type A aortic dissection

Arterial cannulation site	Advantages	Disadvantages
Femoral	1. TEE can guide procedure. 2. Median sternotomy not required. 3. Procedure is very rapid. 4. Surgeon has familiarity. 5. May improve renal perfusion.	1. Requirement for additional access for anterograde cerebral perfusion 2. Concern for increased atheroembolism risk 3. Potential for false lumen expansion and poor true lumen perfusion 4. Lower extremity malperfusion risk 5. Increased risk of false lumen cannulation 6. Potential for vascular injury
Axillary/innominate	1. Median sternotomy not required. 2. Anterograde cerebral perfusion readily available.	1. Potential for vascular and nerve injury 2. Time-consuming and technically demanding 3. Upper extremity malperfusion risk 4. Proximal true lumen compression possibly leading to systemic malperfusion
Ascending aorta	1. TEE can guide procedure. 2. Procedure is very rapid. 3. Surgeon has familiarity.	1. Risk of aortic rupture 2. Increased risk of false lumen cannulation 3. Requirement for additional access for anterograde cerebral perfusion
Carotid	1. Procedure is rapid. 2. Median sternotomy not required. 3. Anterograde cerebral perfusion readily available. 4. May benefit patients with cerebral malperfusion.	1. Potential for vascular and nerve injury 2. Proximal true lumen compression possibly leading to systemic malperfusion 3. Theoretical atheroembolism risk
Transapical aortic	1. TEE can guide procedure. 2. Procedure is rapid. 3. Median sternotomy not required. 4. Anterograde true lumen perfusion is reliable. 5. It is a viable option when alterative cannulation sites are unavailable.	1. Contraindicated for severe AS. 2. Exposure with prior cardiothoracic surgery is difficult. 3. May lead to new or worsening AR after cannulation. 4. Requires additional access for anterograde cerebral perfusion. 5. Potential for bleeding at apical cannulation site exists. 6. Cannot cross clamp during the cooling phase before circulatory arrest.

Transesophageal echocardiography is an essential tool in the evaluation and management of patients with aortic dissection. However, the cannulation procedure is arguably the time when the information obtained from TEE is most critical for achieving procedural success and avoiding complications. To guide TAC with TEE, familiarity with the surgical procedure is essential.

The surgeon first begins by identifying the LV apex. Transesophageal echocardiography midesophageal 2-chamber and long-axis views can be used to confirm the surgeon's chosen site for cannulation is at the LV apex and has the correct orientation for cannula/wire advancement along the interventricular septum and across the AV.

Echocardiographers should use the midesophageal long-axis view to guide the wire/cannula advancement along the interventricular septum as it passes through the left ventricle, across the AV, and into the true lumen of the ascending aorta. During advancement of the wire/cannula, it is important to avoid entanglement of the device in the mitral subvalvular apparatus, which may present as new anterior leaflet tethering, new or increased mitral regurgitation, or obvious tangling of the device within chordal structures.^14^ According to expert opinion, the recommended final position of the transapical aortic cannula tip is within the true lumen of the ascending aorta at the level of the sinus of Valsalva or sinotubular junction.^1^^,^^6^ If the cannula tip is too shallow, there is theoretical risk of retrograde flow across the AV from the tip or side flutes of the arterial cannula, leading to inadequate LV venting and decreased true lumen perfusion.

Once the cannula is appropriately positioned, TEE should be used to evaluate for new or persistent AR and mitral regurgitation and perform a hemodynamic assessment, especially in patients with preexisting AS. While AR is a reported complication after TAC and can lead to LV distension and myocardial ischemia, some experts have found that severity of AR may improve after TAC due to improved leaflet coaptation around the cannula.^1^^,^^5^^,^^15^ Additionally, although caution is recommended when performing TAC in patients with AS, most experts only consider severe AS to be a contraindication to TAC.^1^^,^^5^ Theoretical concerns with AS would include embolic phenomenon during cannula placement, inability to place the cannula through a severely stenotic AV, and hemodynamic instability due to further reduction in severely reduced orifice area.

Once CPB is initiated, TEE should be routinely utilized throughout the CPB period to reevaluate for the presence of AR and its effects on LV distention to ensure adequate LV decompression and to confirm appropriate cannula positioning and true lumen perfusion.^1^^,^^3^^,^^5^ After the procedure is finished and CPB is terminated, in addition to a complete TEE examination, it is important to reinspect the native AV and mitral valve to ensure no valvular damage occurred during TAC.

To further increase the likelihood of success and reduce the potential for complications during TAC, we have created a brief outline for TEE guidance of TAC and identification of complications (Figure 3).

**Figure 3:**
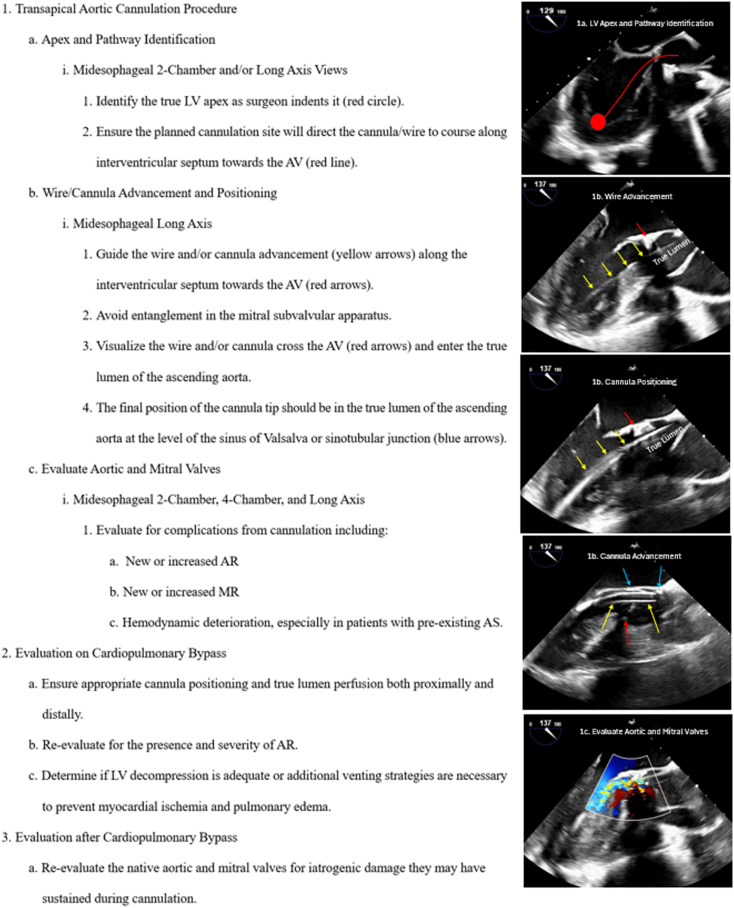
Recommendations for guiding TAC with TEE and identifying potential complications. *MR*, Mitral regurgitation; *MV*, mitral valve.

## Conclusion

Although TAC is still an uncommon cannulation strategy in the setting of an acute type A aortic dissection, it has been shown to be a safe and rapid cannulation method to ensure true lumen perfusion and is an invaluable alterative cannulation method when more common arterial cannulation sites are unavailable. Beyond its value in the diagnosis and management of patients with acute type A aortic dissection, when the decision is made to perform TAC, TEE is an essential tool for both guiding the cannulation process and identifying potential complications. We believe our outline can help others to use TEE to safely and effectively guide TAC in the future.

## Ethics Statement

The authors declare that the work described has been carried out in accordance with The Code of Ethics of the World Medical Association (Declaration of Helsinki) for experiments involving humans.

## Consent Statement

The authors declare that since this was a non-interventional, retrospective, observational study utilizing de-identified data, informed consent was not required from the patient under an IRB exemption status.

## Funding

The authors declare that this report did not receive any specific grant from funding agencies in the public, commercial, or not-for-profit sectors.

## Disclosure Statement

The authors reported no actual or potential conflicts of interest relative to this document.

## Supplementary Data

Video 1 Two-dimensional TEE, midesophageal long-axis (120°) view, demonstrates the bicuspid AV, dilated ascending aorta, and acute type A thoracic ascending aortic dissection with multiple mobile, linear tissues consistent with intimal dissection.

Video 2 Two-dimensional TEE, midesophageal long-axis (137°) view, demonstrates the transapically placed wire originating at the LV apex, crossing the AV, and entering the true lumen of the ascending aorta.

Video 3 Two-dimensional TEE, midesophageal long-axis (137°) view, demonstrates the transapically placed cannula originating at the LV apex, crossing the AV, and entering the true lumen of the ascending aorta.

Video 4 Two-dimensional TEE, midesophageal long-axis (137°) view, demonstrates the transapically placed cannula crossing the AV with cannula tip correctly terminating in the true lumen of the aortic root at the level of the sinus of Valsalva.

Video 5 Two-dimensional TEE, midesophageal long-axis (137°) view with color-flow Doppler, demonstrates an AR jet originating at the site where the aortic cannula crosses the AV.
